# Antenatal prevention of atopic dermatitis associated with food allergies

**DOI:** 10.1186/2045-7022-5-S3-P130

**Published:** 2015-03-30

**Authors:** Vera Revyakina, Elena Berezina, Elena Kuvshiniva, Tatiana Sentsova, Anna Timofeeva, Svetlana Denisova

**Affiliations:** 1 Institute of Nutrition, Moscow, Russia

## 

To establish effect of probiotic *Lactobacillus reuteri *which is used in women in the last pregnancy trimester, prevalence of atopic dermatitis in children of the first 6 months of life. One group of women took *Lactobacillus reuteri* as chewing tablets of probiotic *Lactobacillus reuteri*, with dairy-limited diet excluding walnuts prior delivery. The second group of pregnant women took only proposed diet. Cumulative prevalence of atopic dermatitis was lower in group of children which mothers took *Lactobacillus reuteri* (6.7%) during pregnancy than in group of children of mothers from control group (24.2%). Analysis of the clinical features of disease showed that in children of the main group was observed mild atopic dermatitis (SCORAD amounted to 12,3+0.14 points). In children of the control group was middle and severe of atopic dermatitis (SCORAD 22-52 points, respectively).

The use of *Lactobacillus reuteri* by women in the last pregnancy trimester was positive for general condition and status of gastrointestinal tract. Pregnant women which took *Lactobacillus reuteri* had increased metabolic activity of lactic flora and recovered balance between aerobic/anaerobic microorganisms. Disorders of intestinal microbial balance in pregnant women in control group were not recovered to the end of pregnancy.

